# Probing the influence of mutations on FUS condensates, one molecule at a time

**DOI:** 10.1038/s42003-020-01560-6

**Published:** 2021-01-04

**Authors:** Krishnananda Chattopadhyay

**Affiliations:** Structural Biology and Bioinformatics, CSIR-Indian Institute of Chemical Biology, 4 Raja SC Mullick Road, Kolkata, 700032 India

## Abstract

Protein aggregation and phase separation appear to play important roles in diseases like amyotrophic lateral sclerosis (ALS) and frontotemporal lobar dementia (FTLD), but the interplay between different participating molecular events-which may facilitate or inhibit one another-can be difficult to study by conventional ensemble methods. In a recent study, Kevin Rhine and co-workers make use of point mutations to demonstrate the contrasting behaviour of condensates arising from Glycine and Arginine FUS mutants using single molecules fluorescence measurements.

While being united may have helped humankind to stand on its feet, separation may be key for some protein molecules—particularly when it comes to a number of neurodegenerative diseases. It is known that protein molecules in some of these diseases may club together forming fibrillar aggregates, which wreak havoc on the human nervous system. Recent research has shown that ailing proteins may have a way to rescue themselves through a mechanism called phase separation. While the processes of aggregation and phase separation may have strong implications for diseases such as ALS and FTLD, these can be modulated by multiple events in a heterogeneous macromolecular interacting landscape, in which the quantification of the contribution of an individual component—while necessary for successful understanding of the disease biology—is difficult and often impossible by conventional ensemble measurements (which provide only an average snapshot of thousands of participating events). Scientists are often puzzled about what roles (if any) phase separation play to rescue the proteins from travelling towards the pathway of aggregation. It is also not understood if the available disease mutants have anything at stake in these processes, and, if they do, to what extent. One example of such a system comes from a protein called Fused in Sarcoma (FUS), which undergoes rapid, physiologically reversible phase separation to form biomolecular condensates. Kevin Rhine and co-workers at Johns Hopkins University make use of point mutation and single-molecule fluorescence measurements to demonstrate the contrasting behaviour of condensates arising from Glycine and Arginine FUS mutants in ALS/FTLD^1^.

Pathological inclusion of FUS is hallmark for ALS and FLTD. FUS, like many other RNA binding proteins, possesses intrinsically disordered regions (IDRs), and is prone to aggregation. Protein-protein and protein-RNA interactions play crucial roles in modulating LLPS behaviour of FUS. While there are more than 70 ALS/FTLD-linked mutations in FUS, arginine (R) mutants have been found to have defective interactions with RNA and glycine (G) mutations form solid-like condensates. Rhine et al. have used single-molecule spectroscopy and microscopy to investigate how G- and R-mutants modulate the phase behaviour of wild type (WT) FUS and the physical nature of FUS condensates, including the difference in droplet fluidity, size and co-localisation.

The following are some of the salient features:

- FUS G-mutants prevent association with WT FUS within the droplets, which exclude each other from interacting with the same RNA. In contrast, R-mutants physically interact with WT FUS forming co-condensates. As a result of this mutual inclusion, WT recovers R mutant defects by ameliorating RNA binding.
- Mutational analyses reveal that the position of the mutation and the bulkiness of the amino acid substitution play important role in the molecular defects observed for the ALS/FTLD-linked G156E mutation. Bulky amino acid substitution at Gly-156 (but not at Gly-154) drives the formation of dynamically arrested condensates.

A possible physical explanation for the contrasting behaviour of G- and R-mutants has been provided, using the stickers-and-spacers model. The change of glycine (spacer) to glutamate at position 156 would lower percolation concentration by replacing a spacer residue with a bulky amino acid. As a result, the Glycine mutants would make favourable crosslinks with other Glycine mutants and not with WT protein. Rhine et al. show that large-scale replacement of glycine residues in FUS decreases the mobility of the resulting condensates, highlighting on the role of glycines as important spacers that lower gelation propensity. They further postulate that spacers within WT protein are more solvated and are therefore unable to interact with Glycine mutant proteins.

This study emphasises further the importance of point mutations in FUS and its RNA binding that modulate the LLPS behaviour. The use of single-molecule measurements offers additional insights into the complexity of aggregation-LLPS landscape, which may be needed to understand disease progression in vitro and inside cell and development of treatments against ALS/FTLD.
